# Hyperspectral Features of Oil-Polluted Sea Ice and the Response to the Contamination Area Fraction

**DOI:** 10.3390/s18010234

**Published:** 2018-01-15

**Authors:** Bingxin Liu, Ying Li, Chengyu Liu, Feng Xie, Jan-Peter Muller

**Affiliations:** 1 Environmental Information Institute, Navigation College, Dalian Maritime University, Dalian 116026, China; gisbingxin@dlmu.edu.cn; 2 Key Laboratory of Spatial Active Opto-electronic Technologies, Shanghai Institute of Technical Physics, Chinese Academy of Sciences, Shanghai 200083, China; liuchengyu@mail.sitp.ac.cn (C.L.); xf@mail.sitp.ac.cn (F.X.); 3 Mullard Space Science Laboratory, Department of Space and Climate Physics, University College London, Holmbury St. Mary-Surrey RH5 6NT, UK; j.muller@ucl.ac.uk

**Keywords:** sea ice, oil spill, spectral features, area fraction

## Abstract

Researchers have studied oil spills in open waters using remote sensors, but few have focused on extracting reflectance features of oil pollution on sea ice. An experiment was conducted on natural sea ice in Bohai Bay, China, to obtain the spectral reflectance of oil-contaminated sea ice. The spectral absorption index (SAI), spectral peak height (SPH), and wavelet detail coefficient (DWT d5) were calculated using stepwise multiple linear regression. The reflectances of some false targets were measured and analysed. The simulated false targets were sediment, iron ore fines, coal dust, and the melt pool. The measured reflectances were resampled using five common sensors (GF-2, Landsat8-OLI, Sentinel3-OLCI, MODIS, and AVIRIS). Some significant spectral features could discriminate between oil-polluted and clean sea ice. The indices correlated well with the oil area fractions. All of the adjusted *R*^2^ values exceeded 0.9. The SPH model1, based on spectral features at 507–670 and 1627–1746 nm, displayed the best fitting. The resampled data indicated that these multi-spectral and hyper-spectral sensors could be used to detect crude oil on the sea ice if the effect of noise and spatial resolution are neglected. The spectral features and their identified changes may provide reference on sensor design and band selection.

## 1. Introduction

The ice-covered area in the Arctic is declining annually because of global warming. In particular, the shrinking of the ice area during summer is considered as a serious threat. A large amount of oil, natural gas, and other resources are buried under the seabed and land in the Arctic. The United States, Russia, and other countries conduct deep-sea drilling for oil and gas resources in this region [1,2]. In the northern waters of China, large oil fields, such as Liaohe and Dagang, are distributed in the Bohai Sea and its surrounding area. Freezing conditions occur during winter, which experiences a strong and stable Mongolian cold-core high. The sea ice formed in this region constitutes the southern margin of the frozen sea area in the northern hemisphere. Ice monitoring in the Bohai Sea shows that the annual glaciation in the Bohai Sea is approximately three to four months long. The Liaodong Bay has the longest glaciation, followed by Bohai Bay. Offshore shallow water areas are significantly impacted by sea ice formation, and offshore oil production in the Jidong, Dagang, and Liaohe Oilfields is mainly concentrated in those areas.

The presence of sea ice causes severe harm to oil production and transportation, including damage to passing ships, oil platforms, and oil pipelines, which often leads to oil spills [2]. The opening of the Arctic route, resource development, and the rise in the number of naval vessels in the ice areas in the North China Sea have certainly increased the possibility of oil spills in the sea ice area [3,4,5]. These activities have increasingly become the focus of maritime and marine regulatory authorities [6]. The clean-up of an oil spill is more difficult in ice areas than in ice-free open water. Oil spills in sea-ice covered areas have extensive impacts, and the consequences are severe [7,8]. For example, in 1989, the Exxon Valdez tanker spilled approximately 37,000 t of crude oil into Alaskan waters, affecting a 1900 km coastline. Exxon hired 11,000 workers and spent nearly a year for the clean-up, but the impact of the spill on the marine environment remains.

In recent years, remote sensing technology has led to considerable advances in research on oil spill detection and area inversion [9,10], but only a few studies have focused on using remote sensing techniques to monitor oil spills in ice-covered sea waters [11]. Most existing studies are from countries in North America and Northern Europe. In one study, researchers conducted laboratory and field trials in the Beaufort Sea in Northern Canada to test a variety of sensors and technologies [12]. Researchers in Canada and Norway have also started exploring the availability of a new generation of ground-penetrating radar, sonar, and methane detectors [4]. ExxonMobil has begun experimenting with nuclear magnetic resonance-based airborne detection systems [13]. Nine international oil and gas companies, including BP and Shell, supported the Joint Industry Program (JIP), which aimed to further improve responsiveness to oil spills and to test the capability of existing oil spill emergency technology and equipment to operate in polar regions [14]. Finally, Dickins et al. [5] conducted airborne oil spill monitoring experiments in Arctic waters. These researchers observed the experimental sea area using synthetic-aperture radar (SAR) and side-looking airborne radar (SLAR) and were able to identify ships and ship wakes. However, they could not identify features pointing to the presence of oil. Therefore, SAR is the most effective means of oil spill monitoring in the open waters, but not in the sea ice area, because it cannot identify oil on sea ice.

The identification of oil spills by visible-near-infrared (VIS-NIR) remote sensing technology is less dependent on the sea surface roughness than radar, mainly due to the different reflectivity and emissivity of various surface features. Therefore, optical remote sensing data can successfully identify oil spills on the sea ice area under adequate lighting conditions. Multi-spectral and hyperspectral remote sensing data are extensively used for open water oil slick monitoring, but rarely have they been applied to oil spill monitoring in the sea ice area. Praks et al. used an airborne imager spectrometer to monitor the oil spill in an ice area in Finnish waters in April 2003. They analysed the spectral characteristics of sea ice, sea water, and oil-contaminated sea ice, and proposed an oil spill recognition algorithm for the sea ice area [15]. Despite the experiments conducted by Parks et al. [16] and Chao et al. [17], research on optical remote sensing monitoring of oil spills in sea ice areas is still inadequate. The offshore sea ice is not as clean as that of an indoor pool, and thus, their optical properties differ markedly. Accordingly, it is likely that the results of indoor experiments may not be applicable to the natural offshore environment. The spectral features of the field experiment match the natural circumstances more closely. The existence of sediment, salt, and water makes it more difficult to eliminate false targets from oil-polluted areas [18]. Furthermore, research on spectral reflectance characteristics of oil pollution in visible-near-infrared bands under sea ice conditions is lacking [19]. 

The goal of this study is to extract the spectral reflectance characteristics of oil-contaminated sea ice in the VIS-NIR band and to explore the correlation of these spectral characteristics with the oil area fraction. In this study, an experiment was conducted on natural sea ice in Bohai Bay, China. The spectral reflectances of oil-polluted sea ice with different oil area fractions and that of false targets were obtained. Specific indices, namely, spectral peak height (SPH), spectral absorption index (SAI), and wavelet detail coefficient (DWT d5), were analysed. The correlation between these parameters and the oil area fraction was analysed through stepwise multiple linear regression (SMLR). The spectra were resampled to GF-2, Landsat8-OLI, Sentinel3-OLCI, MODIS, and AVIRIS to evaluate the potential of these sensors to detect oil on sea ice. 

## 2. Materials and Methods

### 2.1. Experimental Conditions

This experiment analysed the Bohai Bay waters of Huanghua, China, on 1–2 February 2013, during a field survey designed to obtain the spectral reflectance data of polluted sea ice in a natural sea ice environment. The thickness of the sea ice in the experimental area was approximately 20 cm. The experiment was conducted under sunny, cloudless weather conditions to ensure adequate and stable lighting. The mean site temperature was −6 °C.

### 2.2. Materials

The crude oil used in the experiment was sourced from the local Dagang Oilfield (Huanghua, China) and had a pour point of 20 °C. The crude oil was thus heated to normal crude oil storage temperature to retain its flow characteristics. The oil was poured onto the sea ice 2 h prior to the measurements that were taken, before the interface between the ice and oil froze again.

**Figure 1:**
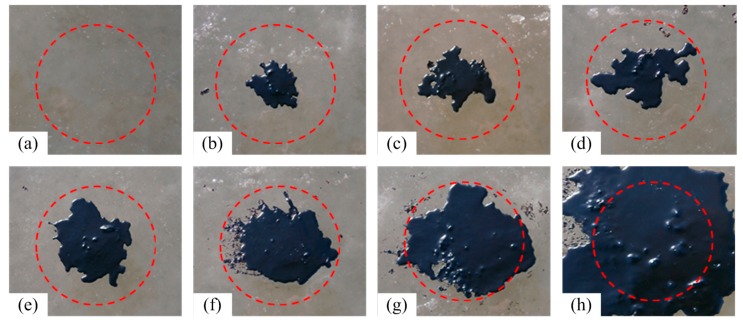
Schematic of oil pollution (the red circle represents the observation range of the spectrometer probe). (**a**) is the non-polluted sea ice; the proportion of the oil-polluted area in (**b**–**h**) is 23.92%, 31.65%, 41.56%, 52.95%, 60.79%, 81.88% and 100.00% respectively.

Eight sample areas, including one clean sea ice sample area and one sample area completely covered by crude oil, were set up such that the proportion of the oil-polluted area within the observation range of the detector increased gradually from 0% to 100%. Note that the extent of the oil slick area discussed in this paper could not be increased proportionally given the poor controllability of the diffusion area of the oil slick under low-temperature conditions (Table 1, Figure 1). The method used to calculate the area is described in Section 2.4.

**Figure 2:**
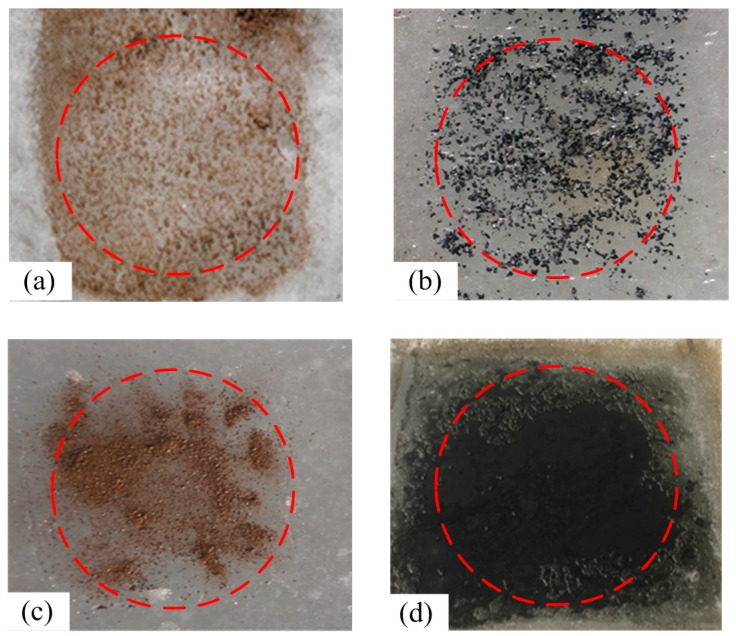
Schematic of the false targets. Sea ice covered by sediment (**a**); coal dust (**b**); and iron ore fines (**c**). The ice was melted, resulting in a dark melt pool (**d**).

The other four sample areas were also set up to simulate the false targets caused by the melt pool and other substances. Coal dust, iron ore fines, and sediment were spread on the surface of the sea ice to imitate pollution under natural circumstances (Figure 2a–c). A chosen area was heated to melt the sea ice. This area was used as the melt pool sample (Figure 2). 

### 2.3. Instrument and Spectral Measurement

**Figure 3:**
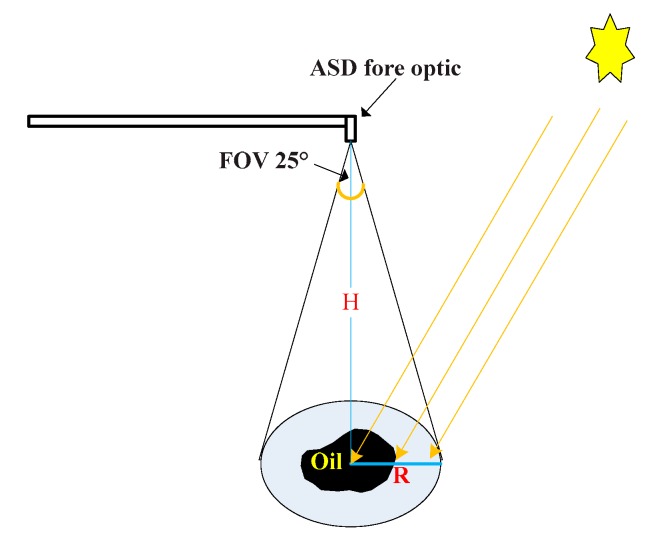
Observation geometry.

An ASD FieldSpec^®^3 (ASD Inc., Longmont, CO, USA) spectroradiometer was used in the study. Its spectrum range was 350–2500 nm. It had sampling intervals of 1.4 nm (350–1000 nm) and 2 nm (1000~2500 nm), and the probe field angle was 8°. A whiteboard was used as the standard reference board for diffuse reflection matching with the spectroradiometer. The viewing angle of the spectrometer was 25°, the observed zenith angle was 0°, the probe height H was approximately 45 cm, and the observation range comprised a circular area, with a radius of approximately 9.98 cm (Figure 3).

The scope of the observations included bare ice and pollutants. For the oil-polluted sample areas, the proportion of areas that were covered by bare ice and oil slick were different. The radiation of the reference whiteboard was first measured during the spectrum measurement, and then each sample was measured sequentially for approximately 40 s. Finally, the radiation for the reference whiteboard was measured again. The measurements were repeated thrice. The duration of the experiment was approximately 30 min, during which the solar elevation angle changed by less than 2°. The area and thickness of the oil slick were re-measured after completing the reflectance spectrum measurement. The change was found to be negligible, that is, the oil slick diffusion rate was minimal over the course of the experiment.

The spilled oil slick on the surface of the sea ice was collected and transported to the laboratory for disposal after the experiment to avoid polluting the marine environment.

### 2.4. Area Calculation

The crude oil diffused in irregular shapes after being spilled on the ice, causing difficulties in calculating the area polluted by the oil. The following method was used in this study to measure the diffusion area of the crude oil accurately.

First, six groups of circular reference areas (the actual area is denoted as $S_{ract-i}$, i=1,2,…6) with a known radius on the ice surface were set. Then, the photos were captured using digital cameras from a height of 45 cm above the ice, that is, the observation geometry corresponded to the spectral measurement geometry. Next, photos of each spectral measurement sample area were also captured using the same observation geometry. Finally, the captured images were imported into ArcGIS, and the area of each reference area $S_{ract-i}$, (i=1,2,…6) was calculated using the area calculation function. The average fraction of the calculated area to the actual area of each reference circle was calculated using the formula shown below in Equation (1).

(1) $$C=average(\frac{S_{ract-i}}{S_{rcal-i}}),\;i=1,2,\ldots 6$$

The area polluted by oil $S_{ract-i}$, (i=1,2,…6) was calculated using the ArcGIS area calculation function, and the actual oil-polluted area was calculated according to Equation (2).

(2) $$S_{oact-i}=C\times S_{ocal-i},\;i=1,2,\ldots 6$$

### 2.5. Data Processing and Analysis

#### 2.5.1. Reflectance Calculation

The reference panel was used to simulate the Lambertian body when the reflectance measurement was performed in the field. The reflected radiance of the reference panel is defined as the radiance reflected by the reference panel in the 2*π* space. Experimentally, the Hemispherical Direction Reflectance Factor (HDRF) was observed and can be expressed by Equation (3), as follows [20]:

(3) $$R(\underline{\cap },\theta_{v},\phi_{v},\lambda )=\frac{dL(\theta_{v},\phi_{v},\lambda )}{dL_{R}(\lambda )}\times R_{R}(\lambda )$$

where $R(\underline{\cap },\theta_{v},\phi_{v},\lambda )$ is the HDRF in a given direction and $L_{R}(\lambda )$ is the observed radiance of the reference panel, which is considered a Lambertian body in this study. Thus, its directional hemisphere reflectance is independent of the viewing direction. $dL(\theta_{v},\phi_{v},\lambda )$ is the reflected radiance of the sea ice observed in a certain direction, and $R_{R}(\lambda )$ is the directional hemisphere reflectance of the reference panel. In vertical observation geometry, $R(\underline{\cap },\theta_{v},\phi_{v},\lambda )$I s commonly known as the reflectance R(λ).

The initial measurement obtained by the FieldSpec 3 spectrometer is the reflected radiant energy value L, generally expressed by the digital number (*DN*) value. The method for calculating the oil pollution reflectance is shown in Equation (4) [21]:

(4) $$R_{oil}(\lambda )=\frac{DN_{sample}(\lambda )}{DN_{R}(\lambda )}\times R_{R}(\lambda )$$

where $DN_{sample}(\lambda )$ is the *DN* of the sample, and $DN_{R}(\lambda )$ is the *DN* of the reference panel.

In the field reflectance measurements, the noise of the data is large due to the influence of water vapour absorption [22]. Therefore, the bands with poor signal-to-noise ratio should be removed before the next step, which in this case, includes the bands at 1330–1430, 1800–1950, and 2300–2500 nm. The median filter was applied to the reflectance spectral curve to reduce the impact of noise on the spectral characteristics, and the filter window size was set to 5.

#### 2.5.2. Statistical Analysis and Modelling

Spectral reflectance data show strong co-linearity between wavelengths, which results in data redundancy. In this study, dimensional reduction of the measured data was accomplished by feature selection [23]. The bands with significant characteristics in the spectral reflectance data were selected as the inputs for the SAI and SPH. The spectral data were analysed by discrete wavelet analysis (DWT), and the wavelet detail coefficients at specific wavelengths were selected [24]. The SMLR method was used for the modelling.

Several spectral indices are used to describe the characteristics of the reflection spectrum, such as spectral absorption depth, absorption width, and SAI [25,26]. The SAI and SPH were the spectral indices in this study.

**Figure 4:**
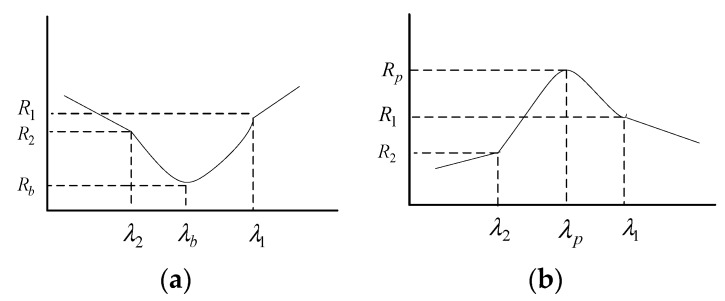
Schematic of spectral absorption index (SAI) (**a**) and spectral peak height (SPH) (**b**).

The SAI is defined according to Equation (5), as

(5) $$\mathrm{SAI}=\frac{dR_{1}+(1-d)R_{2}}{R_{b}},\;d=\frac{\lambda_{b}-\lambda_{2}}{\lambda_{1}-\lambda_{2}}$$

where $\lambda_{1}$, $\lambda_{2}$ and $\lambda_{b}$ are the corresponding wavelengths of the shoulder and bottom of the absorption spectrum, and $R_{1}$, $R_{2}$ and $R_{b}$ are the corresponding reflectances (Figure 4a).

The SPH is defined according to Equation (6) as

(6) $$SPH=R_{p}-\frac{R_{1}+R_{2}}{2_{}}$$

where $R_{1}$ and $R_{2}$ are the reflectances at the lower sides of the reflection peak, and $R_{p}$ is the reflectance at the peak (Figure 4b).

The DWT method can not only separate signal from noise, but can also highlight the characteristics of the original signal. The location of the extreme value point of the wavelet coefficient is the position of the singularity of the original spectral signal, and the singularity of this point is significant when the absolute value of the wavelet coefficient is large [27]. Based on tests of different wavelet functions and decomposition levels, this study selected the db4 wavelet function to decompose the reflectance data into five layers. After decomposition, the fifth-layer detail coefficient (d5) was calculated to extract the reflectance spectral characteristics.

The SAI, SPH, and wavelet detail coefficient datasets were used to build the model. The construction of the model used 75% of the data in each data subset, while the other 25% was used as test data for validation. This study used SMLR for model building [28] for these data subsets, where the oil area fraction is the dependent variable and the confidence interval is 95%. All of the built models were applied to the validation dataset.

#### 2.5.3. Spectral Resampling

In order to simulate the reflectance spectra of images from airborne or spaceborne sensors, we resampled the measured spectra to the spectral resolution of some commonly used sensors [29]. First, the wavelengths and full width at half maximum (FWHM) information of five commonly used sensors, namely GF-2, Landsat8-OLI, Sentinel3-OLCI, MODIS, and AVIRIS, were obtained. With this information, we used the spectral resampling module in ENVI to resample the spectra [30]. 

## 3. Results

### 3.1. Reflectance Spectra 

**Figure 5:**
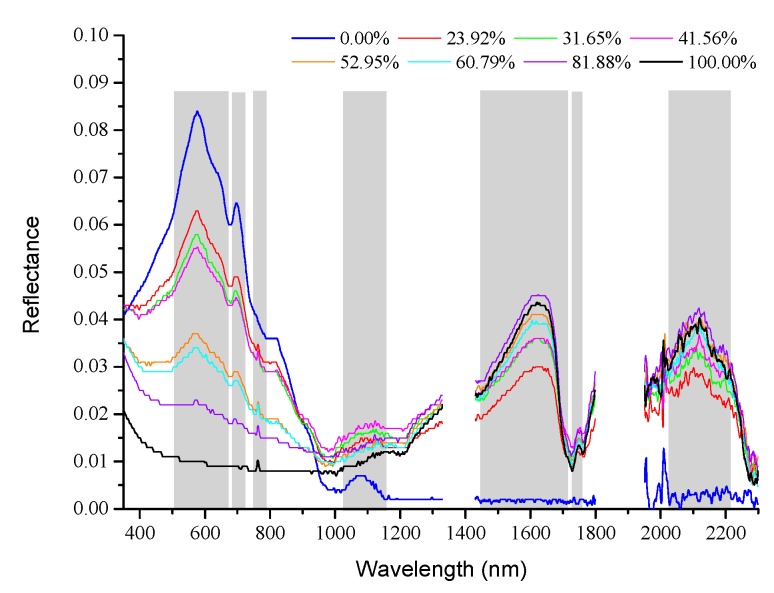
Reflectance spectra of ice with different oil-polluted area fractions (expressed as percentages in the legend).

Figure 5 depicts the spectral reflectance curves for different oil area fractions (0–100% oil-polluted area). The reflectance spectrum of pure sea ice has two large reflection peaks (578 and 1085 nm) and two small reflection peaks (700 and 820 nm). The reflectance of sea ice is quite low (less than 0.005) at bands greater than 1200 nm. There are two reasons for the low reflectance of sea ice. First, the sea ice is very thin. As the research of LI et al. [31] showed that thinner sea ice had a lower reflectance. Second, the upper layer of the sea ice melted and reformed, resulting in conditions less air bubbles in the sea ice. According to the research of SUN [32], less bubble ice has a lower reflectance. The reflectance of crude oil (area fraction 100%) is fairly constant below 1200 nm (the maximum value of reflectance is 0.02 and the minimum value is 0.009). A small reflection peak exists at 760 nm. The reflectance of the oil slicks in the shortwave infrared bands increases gradually, with two distinct reflection peaks at 1620 and 2118 nm, and one absorption valley near 1720 nm. The reflectance spectra of the samples with an oil-polluted area fraction between 0% and 100% fall between those of clean sea ice and pure crude oil.

**Figure 6:**
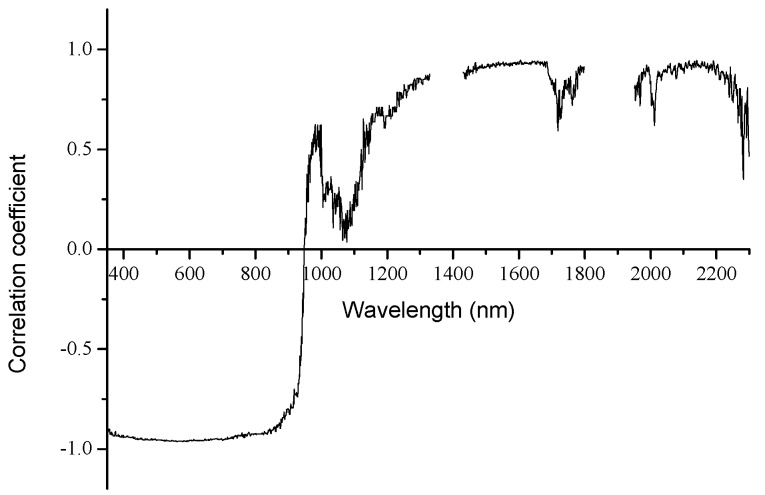
Correlation coefficient for the linear regression of spectral reflectance on the oil-polluted area fraction plotted against wavelength.

The correlation coefficients between the reflectance and the oil area fraction were calculated (Figure 6). The result shows that over 81.3% of the spectral range, the correlation coefficients are larger than 0.9. The increase in the oil-polluted area fraction has a strong linear correlation with reduced spectral reflectance in the VIS-NIR, and increased spectral reflectance in the shortwave infrared bands. Similar to the reflection in the shortwave infrared bands, the absorption of the samples in the visible bands increases gradually with the increase in the oil-polluted area fraction. The solar radiation cannot pass through because the oil slick is thick, and the reflection of water under the ice and the scattered energy of particles in the ice cannot pass through the oil slick. The reflection characteristics of the sea ice–crude oil mixed pixels can also be described by the linear mixing of pixels.

**Figure 7:**
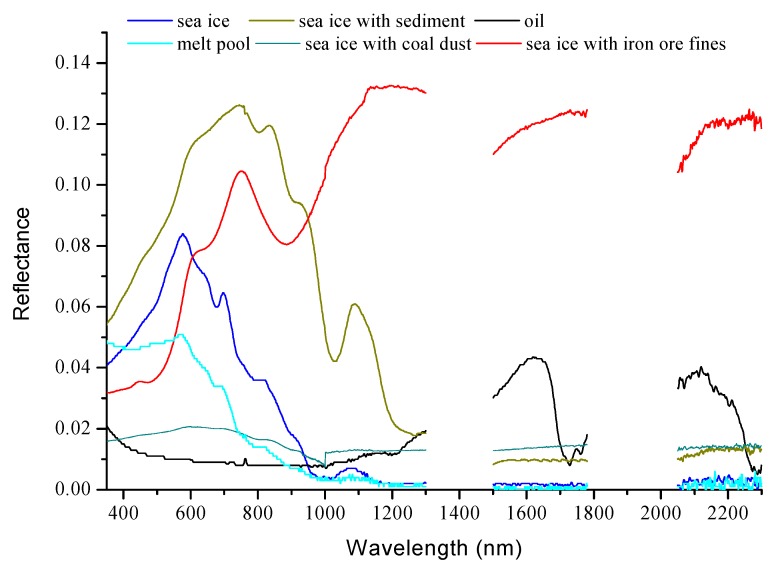
Reflectance spectra of ice with sediments, coal dust, iron ore fines, and that of melt pool.

Figure 7 shows the reflectance spectra of sea ice covered by sediment, coal dust, iron ore fines, and the melt pool. The sea ice covered by sediment reflects more energy than clean sea ice and oil in the visible and short near-infrared ranges. Two reflectance peaks can be noted at 780 nm and 1100 nm, which are obviously different from those for oil. When compared to the clean sea ice and oil, the reflectance of the iron ore fines-covered sea ice appears to be higher at the near-infrared range. The reflectance peak at 780 nm and absorption value at 1040 nm differ from the corresponding values for oil. The sample with coal dust appears darker than the sea ice, but brighter than oil. Its reflectance in the visible band range changes slightly with a relative peak at 610 nm. The melt pool shows a reflectance spectrum similar to that of water. The reflectance decreases with the increase in wavelength. At ranges of 1500 to 2300 nm, the melt pool and oil show lower and much higher reflectances, respectively.

### 3.2. Wavelet Detail Coefficients of Oil on Sea Ice

**Figure 8:**
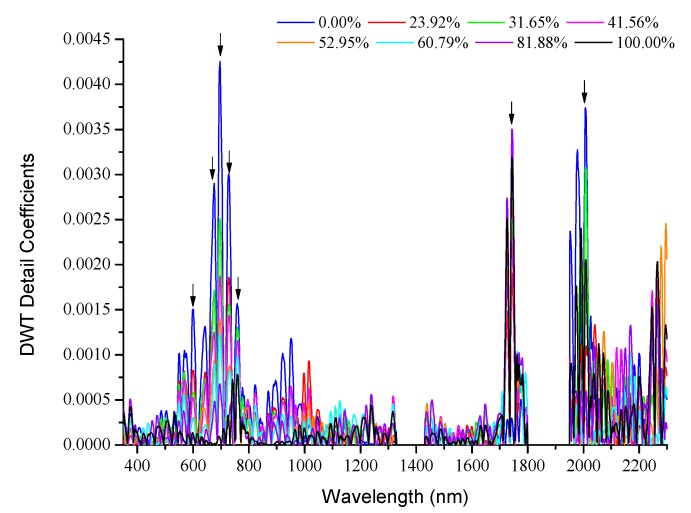
Absolute value of the fifth-level detail coefficient of the wavelet-transformed reflectance spectra of samples with different oil-polluted area fractions (expressed as percentages in the legend).

The db4 wavelet was selected to process the spectral reflectance data. Figure 8 displays the variation of the absolute value of the fifth-level wavelet detail coefficients with wavelength after discrete wavelet transform. The relationship between the wavelet detail coefficients of the reflectance spectra and the oil area fractions was analysed. The wavelengths with large wavelet detail coefficients corresponded to the extreme points of the reflectance spectra. Furthermore, the numerical values are linearly related to the changing trend of the oil area fraction, although the correlation is weaker than that for the spectral reflectance data.

### 3.3. Modelling

The presence of oil pollution significantly reduces the spectral reflectance of sea ice and weakens its spectral characteristics, such that only the spectral features of crude oil remain prominent. This study selected the bands which displayed significant changes in spectral characteristics as a function of oil-polluted area fraction (Figure 5 and Figure 8) for SMLR model analysis.

The SPH and SAI of the reflectance spectra were calculated. The following bands were finally selected for SMLR model analysis: 507–670, 678–707, 756–771, 1028–1153, 1430–1725, 1627–1746, and 2026–2204 nm. The DTW detail coefficients at 601, 676, 697, 729, 758, 1744, and 2008 nm were computed for the SMLR model analysis.

Among the candidate bands, the bands used to construct the prediction model of oil-polluted area fraction were as follows: for SPH, 507–670 and 1627–1746 nm; for SAI, 756–771 nm; and, for DWT d5: 676 nm (Table 2.) The four models were all well-fitted, and the SPH model2, which was jointly constructed from two bands, showed the best fit, with an adjusted *R*^2^ of 0.989.

**Figure 9:**
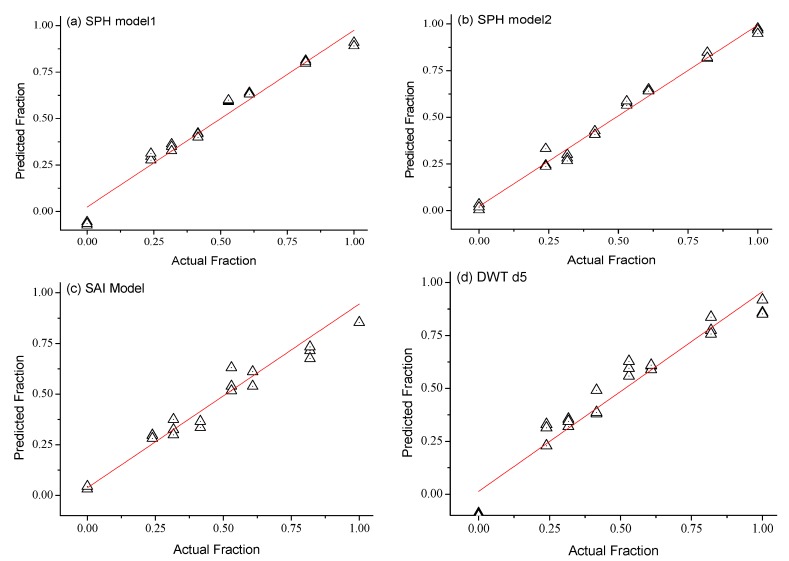
Validation of the models for predicting area fraction of oil on sea ice ranging from 0% to 100%. The adjusted *R*^2^ values for SPH model1, SPH model2, SAI, and DWT d5 are 0.966, 0.986, 0.942, and 0.943, respectively, and the corresponding RMSEs are 0.003, 0.001, 0.005, and 0.005.

The randomly selected verification data consisted of 25% of the spectral reflectance data. The measured value of the oil-polluted area fraction corresponding to the verification data and the value predicted by the model are shown in Figure 9. All four models performed well, with high adjusted *R*^2^ and low RMSE. The SPH model2 performed best.

### 3.4. Potential Capability of Commonly Used Sensors

**Figure 10:**
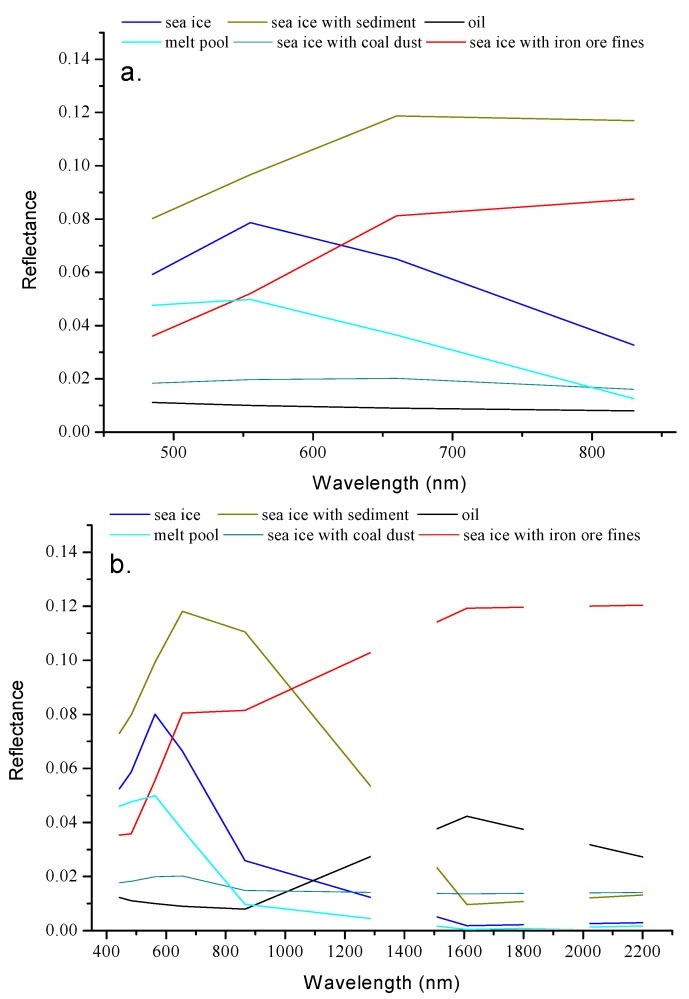

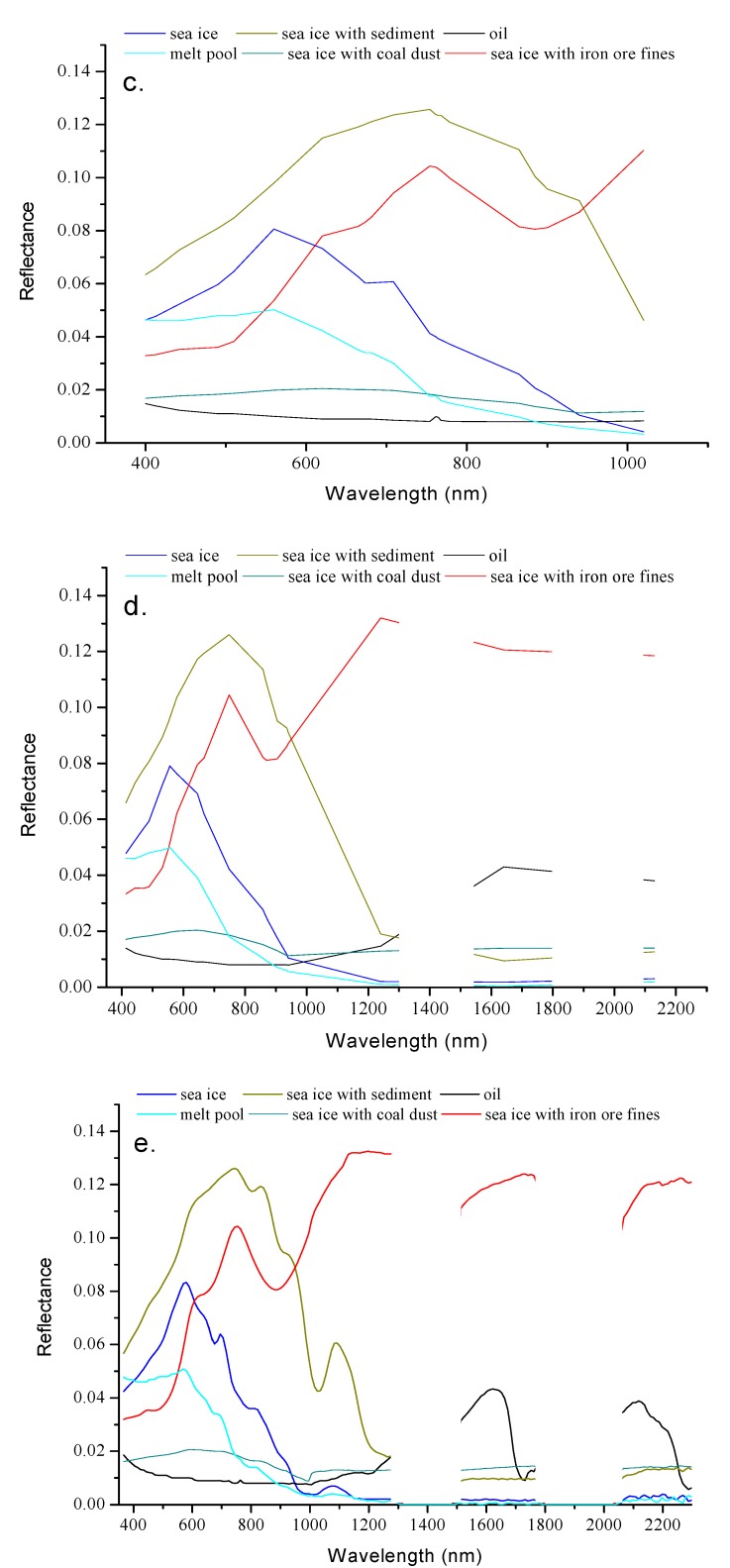
Reflectances of false targets resampled to the band settings of specific sensors: GF-2 (**a**); Landsat8-OLI (**b**); Sentinel3-OLCI (**c**); MODIS (**d**); and, AVIRIS (**e**).

**Figure 11:**
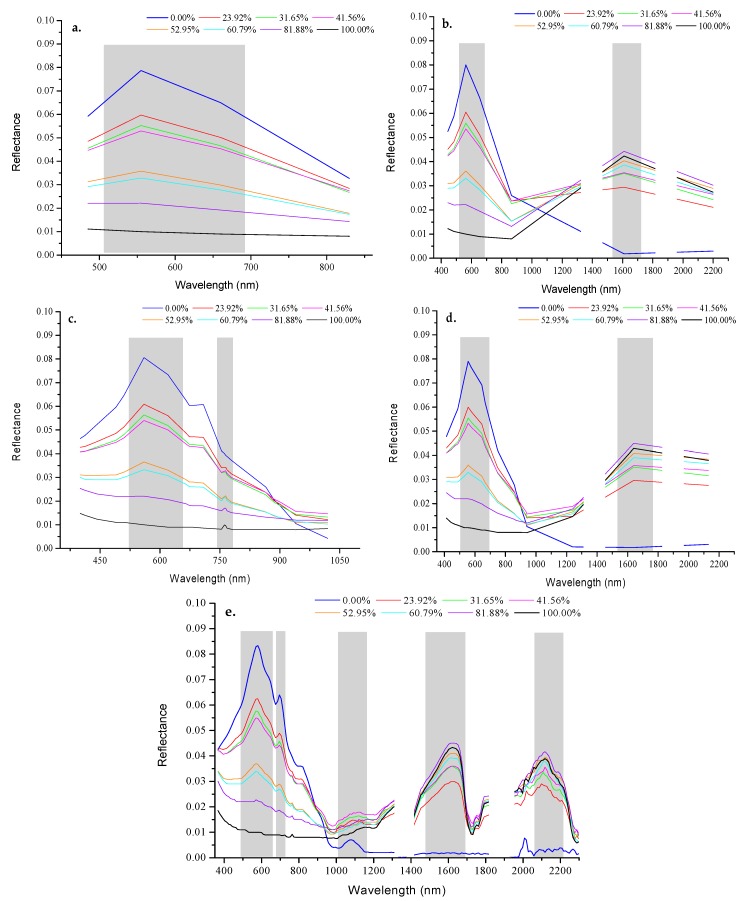
Reflectances of oil-polluted sea ice resampled to the band settings of select sensors. GF-2 (**a**); Landsat8-OLI (**b**); Sentinel3-OLCI (**c**); MODIS (**d**); and, AVIRIS (**e**).

Figure 10 and Figure 11 show the reflectances resampled with the chosen sensors. According to Figure 10, the sea ice with sediment and iron ore fines could be easily distinguished from oil. The coal dust and melt pool could be differentiated from sea ice and oil by the hyperspectral sensors (MODIS and AVIRIS). However, it is quite difficult to distinguish the melt pool and coal dust from the oil-polluted sea ice when the number of bands is insufficient. The number of bands the GF-2 sensor is as low as 4, and thus, the melt pool and coal dust appear to be similar to the oil, while the sediment and iron ore fines appear quite differently when compared to the oil. As Figure 10b shows, bands 6 and 7 of Landsat8-OLI (1560–1660 and 2100–2300 nm, respectively) can potentially discriminate the melt pool and coal dust from oil. Sentinel3-OLCI lacks effective bands to differentiate the oil from the melt pool and coal dust due to its band setting. Figure 10d,e illustrate that MODIS and AVIRIS could provide almost continuous reflectance spectra. All of the spectral differences between the oil and false targets remain. Thus, theoretically, the false targets could be differentiated from the oil.

The simulated spectra in Figure 11 reveal that the diagnostic feature at 578 nm is visible for the five sensors, which means that the bands within the range of 507–670 nm could be widely used to detect oil pollution on sea ice. The GF-2 imagery can only use a single feature to differentiate between oil-polluted and clean sea ice. The Landsat8-OLI and MODIS imageries can take advantage of the reflectance differences at bands within the ranges of 507–670 nm and 1627–1746 nm. The Sentinel3-OLCI imagery captures the spectral characteristics at 507–670 nm and 756–771 nm. Due to its fine spectral resolution, the AVIRIS can collect continuous spectra, such that the AVIRIS imagery shows all of the features that are present in the measured reflectance spectra.

## 4. Discussion

Currently, studies on the VIS-NIR spectral characteristics of oil spills in open waters are relatively abundant [10], but oil spills in ice-covered waters could be identified in a few cases only [9,10]. While the experiment conducted by Chao et al. [17] is similar to the study presented here, they used the average reflectance bands of 1610–1630 nm to estimate the oil-polluted area fraction. Given that the absolute reflectance may vary with the differences in observation geometry or illumination intensity [10], we extracted the spectral characteristics of the sea ice polluted by oil, sediment, coal dust, iron ore fines, and a melt pool. We also analysed the relationship between the spectral features and the oil fraction. Furthermore, we evaluated the potential of five commonly used sensors to detect oil-polluted sea ice and distinguish oil from other pollutants and the melt pool.

The experimental results show that the reflectance characteristics of the VIS-NIR bands can distinguish polluted sea ice from clean sea ice. The presence of crude oil on the ice surface weakens the reflection peak of the clean sea ice at 578 and 700 nm, and a small reflection peak appears near 763 nm. The latter feature could be used to identify the presence of oil pollution on sea ice [33]. For wavelengths that are greater than 950 nm, the reflectance of oil-polluted sea ice is significantly higher than that of clean sea ice, providing another means to identify the presence of oil pollution on sea ice. The attenuation of the reflectance peaks at 578, 700, 763 nm, and in the mid-long wave NIR intensify as the oil-polluted area fraction increases. This result shows that the VIS-NIR reflectance data can be used not only to determine whether oil exists on the ice surface, but also to estimate the oil-polluted area fraction.

Distinguishing oil from sediment and iron ore fines is easy, because their reflectance spectra are quite different, especially in the range of 780 to 1100 nm. The oil could not be differentiated easily from the melt pool and coal dust when the wavelength was less than 1200 nm. The spectra of the melt pool in this range appeared similar to that of water [34]. However, the difference between the oil and coal dust or melt pool became more evident in the 1500 nm to 2300 nm range. 

To that end, we used SMLR to quantify the relationship between the spectral characteristics of the samples at specific bands and the oil-polluted area fractions. The validation of the model using a verification subset of the data denotes that the influence of the oil-polluted area fraction on the spectral features is linear [17]. The oil-polluted area fraction can thus be estimated from the changes in key spectral characteristics. The SPH model2, which was established using the SPH values at 507–670 and 1627–1746 nm, shows the best performance. The SAI-based prediction model, which used the reflection peak at 763 nm, also performed well. The fitting of the wavelet detail coefficient-based model built from the singularity of the reflectance spectra did not perform as well as the other models, mainly because the overall reflectance declines as the oil-polluted area fraction increases, and the changes in the local singularity of the spectra are relatively minimal [27]. 

Analysing the simulated spectra of the selected sensors led us to the conclusion that multi-spectral and hyper-spectral sensors could be used to detect oil pollution on sea ice. All of the examined sensors have the potential to distinguish sediment and iron ore fines from oil. The hyper-spectral sensors are more effective at differentiating oil from the melt pool and coal dust. AVIRIS is the best suited of all the sensors examined here to identify the presence of oil pollution, as well as its area fraction. All of the proposed models in this study could be applied to retrieve oil-polluted area fractions from AVIRIS imagery. 

MODIS, Sentinel3-OLCI, Landsat8-OLI, and GF-2 could be used to detect oil spills on sea ice. When using GF-2 imagery, the SPH model1 could be used to discriminate among various oil coverage area. SHP model1 and model2 are applicable to Landsat8-OLI and MODIS data to calculate the oil fraction within the mixed pixels. The built SHP model1 and SAI model can be used to compute the area fraction of oil-contaminated sea ice from Sentinel 3-OLCI imagery.

In addition to band settings and spectral resolution, environmental noise and spatial resolution should be considered when evaluating the sensitivity of the sensor’s bands to oil pollution on sea ice [35]. Thus, restrictions may still exist for the practical application of the spectral characteristics identified herein. However, the abovementioned spectral characteristics are nonetheless significant as references for sensor design and band selection.

## 5. Conclusions

In this study, the VIS-NIR spectral reflectance characteristics of oil-polluted sea ice were investigated under natural sea ice conditions. The following spectral bands were proposed for quantifying the characteristics of the oil-polluted sea ice surface: 507–670, 678–707, 756–771, 1028–1153, 1430–1725, 1627–1746, and 2026–2204 nm. The spectral characteristics of these bands could not only distinguish between clean and oil-polluted sea ice, but also showed a robust correlation with the oil-polluted area fraction. Besides, we also analysed the spectral differences between oil and sediment, iron ore fines, coal dust, and a melt pool. The findings showed that the reflectance spectra of coal dust and the melt pool were similar with that of oil at the visible bands, but differed significantly in the range of 1500 to 2300 nm. This study calculated the SAI and SPH in order to quantitatively describe the spectral characteristics. DWTs were performed on the reflectance spectra to extract the wavelet detail coefficients at bands with large singularities. These results were then used to calculate the correlation between the spectral characteristics and the oil-polluted area fraction using SMLR. The model using the SPHs in bands 507–670 and 1627–1746 nm, to fit the oil-polluted area fraction, achieved the best results, with the fitting precision reaching 0.986.

These spectral characteristics and their changes can serve to improve sensor design and band selection. In this study, we resampled the acquired spectral reflectance according to the band setting and spectral parameters of five commonly used sensors. We concluded that all of the sensors examined in this study could be used to detect the presence of crude oil on sea ice. However, atmospheric effects, environmental noise, instrument noise, and other factors in the acquisition of real airborne or spaceborne data should not be ignored. 

The validation of the simulation is very necessary and challengeable. However, the examined sensors rarely obtained an image containing the oil polluted sea ice. Currently, we cannot validate by the actual data. This study is the first but very important step in application of remote sensing imagery to detect oil pollution under the sea ice conditions. Further refinement of the method presented here is required to address these issues in future studies. In the future, we plan to use the UAV-borne hyperspectral sensor to conduct the further experiment. Based on the obtained data, we can generate the simulated images using the sensors’ spectral response function. Finally, the generated images could be used to validate the accuracy.

## Figures and Tables

**Table 1:** Proportion of oil-polluted area in each sample area.

Sample	Volume/mL	Oil Area Fraction/%
1	0	0.00
2	30	23.92
3	40	31.65
4	50	41.56
5	60	52.95
6	80	60.79
7	100	81.88
8	200	100.00

**Table 2:** Parameters of the prediction models.

Model Name	Bands/nm	Adjusted *R*^2^
SPH model1	507–670	0.964
SPH model2	507–670 & 1627–1746	0.989
SAI	756–771	0.942
DWT d5	676	0.939
